# State-of-the-art literature review of Recovery College evaluative studies between 2013-2024

**DOI:** 10.3389/fpsyt.2025.1584110

**Published:** 2025-08-04

**Authors:** Catherine Briand, Catherine Vallée, Francesca Luconi, Joanie Thériault, Anick Sauvageau, Julie Bellemare

**Affiliations:** ^1^ Department of Occupational Therapy, University of Quebec at Trois-Rivières, Trois-Rivières, QC, Canada; ^2^ Research Center of Institut universitaire en santé mentale de Montréal, Montréal, QC, Canada; ^3^ School of Rehabilitation Sciences, Université Laval, Quebec City, QC, Canada; ^4^ VITAM Research Center on Sustainable Health, Quebec City, QC, Canada; ^5^ Office for Continuing Professional Development, Faculty of Medicine and Health Sciences, McGill University, Montréal, QC, Canada

**Keywords:** literature review, Recovery College, state-of-the-art, recovery paradigm, chronological analysis

## Abstract

**Introduction:**

Over the past ten years, the Recovery College (RC) practice model has spread at an incredible speed. After ten years of implementation and evaluative research on RC, it seemed worthwhile to analyze the state-of-the-art of these evaluative studies. The aim of this literature review is to provide a systematic analysis answering the questions: 1) Since the first evaluative studies of RC, how have RC studies been developed, implemented and evaluated between 2013-2024? 2) What are the findings and gaps in the studies published between 2013-2024?

**Methods:**

A state-of-the art literature review was conducted with no date limits on peer-review articles in MEDLINE and Scopus electronic databases. The good practice guide for a systematic literature review published by Siddaway et al. was used, in combination with a structured multi-stage process. Endnote, Covidence and NVivo softwares were used to collect relevant evaluative studies, screen them based on blind selection, analyze their content and ensure inter-rater validation. The quality of each study was assessed using the Kmet grids by two independent assessors.

**Results:**

A total of 64 articles published between January 2013 and June 2024 were selected. Analysis of these articles revealed five qualitative clusters. Early articles on the RC focused on implementation stages and lessons (2013-2024). Next, articles focused on perceived benefits, learners’ experience and active ingredients (2014-2024). Articles then moved on to outcomes evaluation (2015-2024) and service utilization and costs (2019-2024). Finally, articles focused on documenting an international scope of the RC and providing a status report and global multicenter comparisons (2019-2023).

**Discussion:**

These groups of articles capture the scope and richness of the studies, but also the progression in study quality over the past 10 years. To keep pace with this progression, future studies need to consolidate outcome measurement and sustainability over time, using models with high statistical power. Thus, they need to move to crossover designs and randomized controlled trials and give preference to multicenter, international studies with high statistical power.

## Introduction

Over the past ten years, the Recovery College (RC) has spread widely and at an incredible speed (1–3). Two hundred and twenty-one RCs are currently operating in 28 different countries and five continents (4). This is probably due to the fact that the RC practice model meets a need for mental health promotion, prevention and intervention that is aimed at everyone.

The first RC was launched in South West London in 2009, and the first publications on the model were published in 2012 (1). RC proposes a health promotion approach where everyone has free access to training courses (in a co-learning workshop format) in mental health (mental health and well-being, mental illness and recovery, combating stigma and living better together) (1, 2). RC training courses are distinguished from other health education models by: (i) the purposive diversity backgrounds of the learners and trainers (people with lived experience of mental health disease, relative of a person with a mental health disease, peer helpers, educational and health professionals, administrative staff, manager and director in educational and health systems, citizens, etc.); (ii) the hybridization and mutual enrichment of knowledge (theoretical, clinical, practical, and experiential) through participatory and discussion-based methods; (iii) the promotion of egalitarian social relationships free of judgment, where speaking out is encouraged (5–7). RC is based on a genuine commitment to co-production and co-learning where lived experience and clinical experience are placed on an equal level, offering learners from diverse backgrounds the opportunity to learn from each other (1, 2). The foundations of RC are based on the fundamental equality of knowledge and human beings, the equitable participation of learners and the experience of egalitarian relationships free of prejudice (including in the RC team i.e., trainers and partner organizations) (5, 8).

RC is aligned with public health policies that advocate the importance of focusing on mental health promotion/prevention, combating stigma and supporting people with mental illness in a recovery-oriented approach (9–12). In 1986, the WHO was already emphasizing the need for a stronger emphasis on self-determination, resilience, literacy, and hope among individuals and communities (13, 14). Even today, societies and healthcare systems are trying to respond to the challenge of implementing mental health promotion and prevention strategies that emphasize individual and community empowerment rather than solely diagnosis and symptom reduction (9, 10, 15). The recovery paradigm is a key aspect of this strategy and forms the basis of the RC (1, 2).

Since the first publications on RC in 2012, several evaluative studies have investigated RC and reported positive outcomes for the learners, trainers and partner organizations involved, at individual, organizational and societal levels. These evaluative studies were captured in some literature reviews (16–18), two of which reported only qualitative papers (19, 20). None of these literature reviews is systematic and has examined all studies from the conception of RC to the present day. These literature reviews reported less than 34 peer-reviewed studies (16–18). After more than ten years of implementation and evaluative research on RC, it seemed worthwhile to analyze the state-of-the-art of these evaluative studies, in order to identify findings and gaps, and to guide future studies.

The aim of this paper is to provide a systematic analysis of the state-of-the-art of peer-reviewed studies listed between 2013-2024. Two research questions guided the analysis: 1) Since the first evaluative studies of RC, how have RC studies been developed, implemented and evaluated between 2013-2024? 2) What are the findings and gaps in the studies published between 2013-2024?

## Methods

### Literature review design

A state-of-the-art literature review design was chosen (21). State-of-the-art literature review provide a time-based overview of the current stage of knowledge about a phenomenon and suggest directions for future research (21). To ensure that this review would meet the best standards of excellence, this state-of-the art literature review followed the guidelines proposed by Siddaway et al. (22) for systematic review. This best practice guide identifies criteria and reflection questions for each stage of the review: scoping, planning, searching, screening, eligibility and studies quality (22). Systematic review attempts to identify, appraise and synthesize all empirical evidence that meets pre-established eligibility criteria to answer a specific research question by a methodical, replicable, and transparent approach (22). Researchers conducting systematic reviews use explicit, systematic methods that are selected to minimize bias, in order to produce more reliable results to inform decision-making (23). This paper also shares, in the second order, similarities with chronological critical review and overview design (24). These approaches analyze the contribution of all published studies in the field (beyond the quality of evidence) in a qualitative and chronological way (24).

### Search strategy

To identify relevant literature, bibliographic search covered two bibliographic databases as recommended by Siddaway et al. (22): MEDLINE and Scopus in May 2023, followed by regular updates in August 2023, January 2024 and June 2024 (by two research assistants AM and LC). The articles included were peer-reviewed articles published from the first RC studies (January 2013) to June 2024. Keywords used were MeSH and text words such as “Recovery College*, Recovery education* center, Recovery College education* centre”. The bibliographic references cited by the included articles and other previously published literature reviews were also examined to identify other relevant articles. The research team’s participation in the international RC community of practice has also helped identify pre-print papers. EndNote bibliographic software (25) was used to extract the articles obtained, sort the references, and leave a record of the selection of papers.

### Inclusion and exclusion criteria

Studies were selected according to the following inclusion criteria: a) evaluative studies (analyzes the components of an intervention) with a qualitative, quantitative or mixed design; b) studies with data collection used to analyze the RC learning center (implementation process, experience, outcomes, etc.); c) primary studies; d) peer-reviewed studies; f) full-text available in English or French. The exclusion criteria are: a) literature reviews (literature reviews were considered only for the validation of primary studies); b) studies not related to the RC; c) studies evaluating a single course with specific topic. This last exclusion criterion was added during the screening process to ensure comparable data for the whole RC learning center.

### Screening process

To ensure inter-rater validation during the screening process, Covidence platform (26) was used. Articles were blindly sorted by two reviewers, a research assistant (AM) and a coauthor (JT), first by title, then by abstract and finally by full text. The first author (CB) was involved as a third reviewer when there was disagreement.

**Figure 1:**
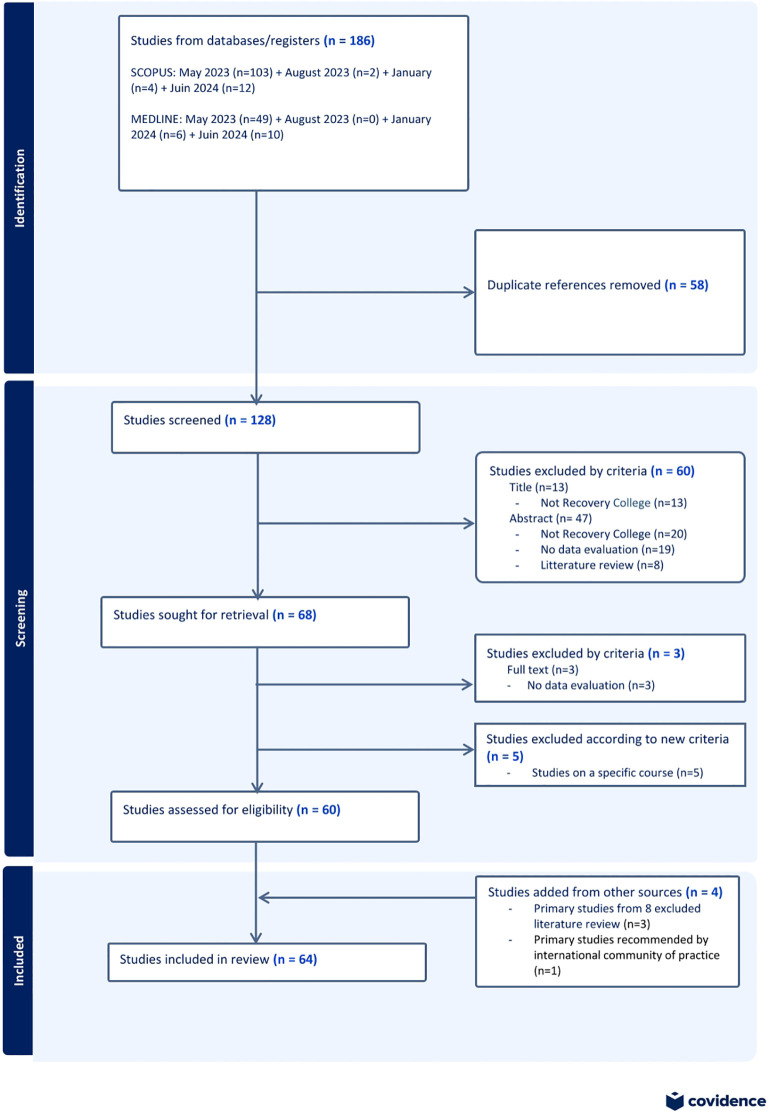
PRISMA flowchart describing the screening process.

Following this process, 64 articles were retained for analysis: 60 articles were found through searches on databases, three articles were added manually from references in literature reviews and another one recommended by the international RC community of practice. **Figure 1** details the screening process via a PRISMA flowchart.

### Quality assessment of the studies

To assess the quality of each study, the quality criteria and grids of Kmet et al. (27) were used by two independent reviewers among the authors (CB, JT, AS, JB) and the research assistants (AM, LC). For each total score with a 2-point disagreement, a discussion was held between the two reviewers, and changes may or may not have been made by each reviewer to move closer to consensus. The mean of the two reviewers’ total scores was used in this study. For studies with descriptive and qualitative designs, the “*Quality Scoring of Qualitative Studies*” evaluation grid (27) was used (10 items for a total score out of 20). For studies with quantitative and mixed designs, the “*Quality Scoring of Quantitative Studies*” evaluation grid (27) was used (14 items for a total score out of 28). Forty-six of the 64 studies achieved a high level of quality, i.e. they met more than 76% of the Kmet et al. (27) criteria (total scores higher than 15/20 or 21/28, depending on the type of study). Kmet et al. (27) cover a range of criteria to support the analysis of the quality of an article: the precision of the objective and research question, the relevance of the design, the methods used to select subjects, the size of the sample, the quality of the analyses, the identification of limitations, etc.

### Data extraction process

For each included article, the following variables were extracted: year of publication, country, design, objectives, study populations, method of analysis, main results and direction for the future. NVivo qualitative analysis software (28) was used to extract information from each article by assigning codes and sub-codes for each variable of interest mentioned above. Coding was carried out by the first author (CB) and validated by two coauthors (JT and JB).

### Qualitative clustering process

Based on coding information and using NVivo qualitative analysis software, articles were characterized according to a finite list of attributes and grouped into qualitative clusters. Attributes were selected to compare articles and enable clustering; with each cluster having similar attributes and being composed of similar articles that are conceptually or methodologically close. Attribute assignment and classification was made by the first author (CB) and validated by three coauthors (CV, JT and JB). The attributes are presented in **Table 1**.

**Table 1:** Set of attributes assigned to each article.

**Attribute 1 – Types of design**	• descriptive method^1^ • qualitative method • quantitative method • mixed method
**Attribute 2 – Number of RCs**	• single • multiple
**Attribute 3 – Target populations/Key informants**	• diverse or specific learners (mental health service users, health professionals, etc.) • diverse or specific trainers • RC staff (managers and trainers) • partner organizations • all (learners, RC staff, partner organizations)
**Attribute 4 – Outcomes**	• implementation lessons • experience and active ingredients • perceived benefits • evaluated outcomes • services use and costs • status report

Attributes have been selected to compare articles with each other and enable clustering. Four attributes were selected to compare studies: the type of design, the number of RCs considered in the study, the target population (or key informants) and the types of findings obtained.

### Within-cluster analysis process

Following the qualitative clustering process, the analysis process was carried out within each qualitative cluster to answer the research questions by the first two authors (CB and CV). Each article was summarized to capture the quality of the design, the variables under study, the target population and the results obtained. The findings reported in this paper identify the contribution of each qualitative cluster’s studies to our understanding of the RC practice model, as well as the next steps required to advance the field of knowledge.

**Table 2** shows the classification of articles by qualitative cluster and by methodological quality. Also, supplementary tables for each qualitative cluster present the articles in chronological order and according to Kmet’s quality criteria.

**Table 2:** Methodological quality of studies by qualitative cluster.

Cluster	Excellent methodological quality (96%-100%)	Very good methodological quality (86%-95%)	Good methodological quality (76%-85%)	Poor methodological quality (75% and under)
**Qualitative cluster I – Implementation lessons (n=12)**	Ali et al. (29) (20/20) Anderson et al. (30) (19.5/20)	McPhilbin et al.(31) (19/20)	Harper and McKeown, (32) (16.5/20) Hopkins, Foster et al. (33) (15.5./20)	Arbour and Stevens, (34) (7/20) Chung et al. (35) (8/20) Dunn et al. (36) (15/20) Frayn et al. (37) (12.5/20) McGregor et al. (38) (12.5/20) Meddings, Byrne et al. (39) (14/20) Zucchelli and Skinner, (40) (8.5/20)
**Qualitative cluster II – Perceived benefits, experience and active ingredients (n=26)**	Doroud et al.(41) (19.5/20) Selbekk et al.(42) (20/20)	Briand et al. (43) (19/20) Crowther et al.(16) (18/20) Dalgarno et al. (44) (18/20) Dalgarno et al. (6) (19/20) Harris et al. (45) (19/20) Khan et al. (46) (19/20) Khan et al. (47) (19/20) Muir-Cochrane et al. (48) (18/20) Oates et al. (49) (18/20) Reid et al. (50) (17.5/20) Thompson et al. (51) (18/20) Whitehead et al. (52) (17.5/20) Zabel et al. (53) (19/20)	Newman-Taylor et al. (54) (17/20) O’Brien et al. (55) (16/20) Perkins et al. (56) (17/20) Sommer et al. (57) (17/20)	Burhouse et al. (58) (12/20) Hopkins et al. (59) (14.5/20) Larsen et al. (60) (8/20) Lucchi et al. (61) (10.5/20) Meddings, Guglietti et al. (62) (14.5/20) Skipper et al. (63) (7/20) Windsor et al. (64) (12/20)
**Qualitative cluster III – Evaluated outcomes (n=13)**	Durbin et al. (65) (28/28) Rapisarda et al. (66) (27.5/28)	Briand et al. (67) (26/28) Hopkins, Pedwell et al. (68) (26/28) Paul et al. (69) (25/28) Yoeli et al. (70) (27/28)	Ebrahim et al.(71) (22/28) Sommer et al. (72) (23/28) Stevens et al. (73) (22/28) Sutton and French, (74) (24/28) Wilson et al. (75) (24/28)	Meddings et al. (76) (17/28) Nurser et al. (77) (15.5/28)
**Qualitative cluster IV – Service utilization and cost analysis (n=5)**		Allard et al. (78) (26.5/28) Bourne et al. (79) (26/28) Cronin et al. (80) (26/28)	Sutton, Lawrence et al. (81) (23/28)	Kay et al. (82) (7/28)
**Qualitative cluster V – Status reports (n=8)**	Hayes, Camacho et al. (83) (28/28) Soklaridis et al. (84) (19.5/20)	Bowness et al. (85) (26.5/28) Hayes, Hunter-Brown et al. (4) (26.5/28)	King et al. (3) (15.5/20) King et al. (86) (22/28) Wolverson et al. (87) (16/20)	Lowen et al. (88) (14.5/20)

## Results

Analysis of the 64 articles revealed five qualitative clusters. These groupings are outlined in chronological order. The first cluster focused on implementation stages and lessons; most of the early papers are within this cluster (2013-2024). The second cluster of articles focused on perceived benefits, learners’ experience and active ingredients (2014-2024). The third cluster focused on outcomes evaluation (2015-2024) and the fourth on service utilization and costs (2019-2024). Finally, the last cluster documented the scope of implementation of the RC practice model internationally and provide status reports and global multicenter comparisons (2019-2023).

### Qualitative cluster I – implementation lessons (n=12)

Twelve articles documented the implementation of a RC (i.e., implementation stages, lessons learned, participation rates, satisfaction levels), with different informants (i.e., learners, RC staff, trainers, partner organizations, etc.) and in different contexts (regular, youth-focused, secure setting, housing instability) (see **Table 2**). These studies took place in UK (n=8), in Canada (n=2), in Australia (n=1) and in Denmark (n=1). Of these articles, five met the criteria of Kmet et al. (27) for qualitative research (29–33). They were published between 2018 and 2024. Despite not satisfying the criteria of Kmet et al. (27), previous descriptive articles represent the history of RC implementation (34–40).

Among the five articles with qualitative designs, two studies focus on general implementation lessons (29, 33). According to these studies, implementing a RC is non-linear process that requires shared understanding of the recovery paradigm and the key principles of the RC (29). Open discussions, about what these principles concretely entail, are necessary to ensure the fidelity to RC, despite the necessary adjustment to the specific context (29, 33). These conversations about how to implement a co-produced and recovery-oriented RC, to share power equally between professionals and people in recovery are identified as essential elements in the desired transformation process (29, 33). Developing a skilled, trained, well-supported and reliable workforce also becomes essential in this quest for quality (33). A critical point in the planning phase is also to determine the funding model and resources brought by partners (29).

Two qualitative studies focus also on the challenges of learner participation (i.e., engagement, support attendance, dropout) (30, 32). These studies address another key principle in the implementation of the RC, i.e., accessibility and inclusion for all. They also identify the main drivers that may limit or impede participation in RC, as some of them may affect more people with lived experience than other learners: 1) external drivers (i.e., transportation, lack of time, physical or mental illness); 2) relational drivers (i.e., relational dynamics between trainers and learners); 3) courses-related drivers (i.e., level of literacy, guidance for recovery stories, balance and space for sharing all types of knowledge) (30, 32). Participation in a RC requires efforts and a true commitment from learners (32).

Finally, one qualitative study examines the impact of Covid-19 in 31 RCs in England and documents the challenges and opportunities in this context (31). The rapid transition to digital delivery and changes in accessibility have accelerated developments in RCs (especially for RCs with decision-making autonomy and agility), positioning them as a more accessible preventative service for people.

This first qualitative cluster of articles “*Implementation lessons*” introduces and explains the RC practice model and provides observations and recommendations for its implementation. Their aim is also to provide an overview of the challenges and facilitators of quality implementation (for both formats, in-person and virtual).

### Qualitative cluster II – perceived benefits, experience and active ingredients (n=26)

Twenty-six articles documented the experience of participating in a RC, the perceived benefits as well as the key/active ingredients that explain these benefits (see **Table 2**). Of these articles, 19 met the criteria of Kmet et al. (27) for qualitative research. These 19 studies took place in UK (n=8), in Canada (n=5), in Australia (n=4), in Norway (n=1) and in Ireland (n=1).

All 19 studies used a qualitative design and, in some cases, included a survey with descriptive analysis. On the other hand, the type of target audience varied across studies. Seven articles examined the combined experience of learners, RC staff and partner organizations (16, 41, 42, 45, 48, 55, 57), including one article focusing on learners and trainers (42). Three articles focused solely on trainer-practitioners (6, 44, 49). Nine articles focused exclusively on learners: five articles on various learners (43, 51–54), one article on learners who are corporate, administrative and clinical staff in the healthcare system (56) and three articles on learners experiencing housing instability (46, 47, 50).

Participation in the RC (whether as learners, trainers or members of a partner organization) resulted in many perceived benefits at individual, organizational and societal levels. At the individual level, the perceived benefits are: 1) better self-awareness, self-confidence, self-worth and empowerment regarding one’s mental health (49–51, 53–57); 2) an increased will to take care of one’s mental health, including self-management and asking for help (43, 48, 50, 51, 53); 3) better interactions and connections with self and others, including the recognition of shared human experiences and the reconsideration of traditional mental health professional roles and worldviews (43, 51, 54, 56, 57); 4) increased sense of belonging and reduced isolation (42, 43, 45, 48, 57); 5) a space for flourishing and growth (41, 45).

In other words, people in recovery were led to engage, to open their horizons to new opportunities, to develop self-advocacy skills, to reclaim their right and agency over their lives (41, 46, 48, 50, 51, 55). Some articles insist on how participation in the RC assists people with lived experience to transition to a more positive identity as citizens (42, 48, 50), find purpose and pursue meaningful life, vocational or educational goals (41, 45, 46, 48, 50, 51, 57). Practical skills were acquired and integrated in daily life by learners with lived experience (53). Mental health practitioners learners reported also some specific outcomes, such as a greater endorsement of recovery-oriented practices, a renewed openness to others and the value of experiential knowledge, the development of a reflective practice on one’s actions and of new clinical skills and a re-engagement and commitment to their work (16, 41, 43, 53, 56, 57).

At the organizational level, the perceived reported benefits included: 1) an increased knowledge dissemination and a deepened understanding of recovery-oriented practices (towards a shift in organizational culture) (16, 53, 56, 57); 2) a redefinition and reevaluation of service user involvement within organizations (16, 44, 57); and 3) reduction of discrimination against workers with experiential knowledge (16, 56).

At the societal level, the perceived benefits documented are: 1) greater partnership and collaboration across organizations, including community organizations (16, 41); 2) change in attitudes toward mental health and stigma reduction (16, 57); 3) a recognition for the need to challenge biomedical views to integrate a more strength-based perspective (16, 56, 57).

Furthermore, sixteen articles described the key and active ingredients of RC, as perceived by learners, trainers and partner organizations. The creation of an easy-to-access, open and inclusive learning space where the importance and contribution of experiential knowledge and co-production is recognized plays as pivotal role (45, 46, 50, 52, 53, 55, 56). Doroud et al. (41) refer to the creation of an “oasis of hope” and inclusion, within a safe space, where one can connect with others differently, in a more meaningful way, within and beyond the RC. Most articles emphasize the importance of co-production and co-facilitation process, where working together in an educational environment to change the relationship between professionals and service users thus reducing the “us and them” distinction (44–46, 51–53, 55–57). This experimentation with a different connection to others leads to changes in the quality and equity of relationships, power dynamics and stigmatizing attitudes, practices and behaviors (16, 44, 52, 54, 57).

This second qualitative cluster of articles “*Perceived benefits, experience and active ingredients*” plays an important role in understanding the “black box” of the RC practice model, and gradually leads to an understanding of the mechanisms of action that enable the perceived benefits.

### Qualitative cluster III – evaluated outcomes (n=13)

Thirteen articles documented the outcomes of participation in RC courses for a variety of learners (see **Table 2**). Of these articles, 11 studies met the criteria of Kmet et al. (27) for quantitative research. These 11 studies come from the UK (n=5), Canada (n=4) and Australia (n=2) and use quantitative or mixed methods design. Six studies documented the outcomes obtained by diverse learners from all backgrounds, while five studies focused specifically on a sub-group of learners: learners with mental health conditions (73, 75), learners with housing instability (65), health professional learners (74) and postsecondary students (69). Most of these studies use pre-post quasi-experimental designs in which the subject is their own control, and standardized questionnaires to assess diverse variables: wellbeing/psychological distress, empowerment, learning goals, personal recovery and goals, health status, social inclusion, opening minds/stigma and quality of life. Of the 11 studies meeting Kmet’s criteria, four pre-post studies published in recent years have been carried out on samples of over 85 learners (65–67, 70).

Several studies suggest significant improvements in wellbeing/psychological distress and empowerment pre- to post- attendance at RC courses (65, 69, 71, 73, 75). Extended participation in RC courses could be associated with better outcomes (72). For instance, Durbin et al. (65) found significant changes in perceived empowerment and mastery only for the subgroup of participants who attended more than fourteen hours of courses in a RC. Three studies support similar results in wellbeing and empowerment with adapted online short format (a 6-hour course) (66, 67) or hybrid format (70). Also, some authors observed significant reduction of anxiety and disclosure/help-seeking after this limited exposure to RC (66, 67).

Some studies suggest that some outcomes may take more time to achieve (72, 75). For example, Sommer et al. (72) reports that attendance rate, number of courses taken, and time spent in RC were the factors that significantly influenced goal attainment. Also, Wilson et al. (75) noted significative improvements in social inclusion of people with lived experience only at 6-month follow-up. Results were less convincing for other outcomes, such as quality of life, recovery or health status for learners with housing instability (65). Regarding open-mindedness and stigma, additional pre-post studies are required (67, 74).

Qualitative data from mixed-design studies show that learners: 1) acquire skills and new strategies to better manage mental health difficulties (70, 71, 73); 2) emerge from isolation and develop a greater sense of connection with their environment (70, 71, 73, 75); 3) gain a renewed sense of hope and confidence (69–71, 73, 75); 4) plan to engage in paid employment or volunteering (72, 73, 75).

This third qualitative cluster of articles “*Evaluated outcomes*” supports the robustness of the RC practice model and highlights the different outcomes of course attendance, regardless of the learner’s background and format of the courses.

### Qualitative cluster IV – service utilization and cost analysis (n=5)

Five articles constitute this qualitative cluster, with four that meet the quality criteria of Kmet et al. (27) for quantitative research (see **Table 2**). Their common interest is to document the use of healthcare services during the participation in RC training courses and to estimate potential cost savings, by using statistical analysis to confirm significant differences in the benefits identified. They address the organizational and societal levels. These studies were realized in the UK (n=3) and in Australia (n=1).

These four studies adopt quantitative designs that use statistical analysis to confirm significant differences in the benefits identified. Sutton et al. (81) compare the employability and the health service utilization of people with lived experience of mental health difficulties and/or substance misuse problems who participated or did not participate in RC courses. Despite a small sample size (n=22 to 31), the results suggest a statistically significant association between course attendance and employment status, where paid or self-employment at follow-up were 4.57 times higher among participants who attended RC courses compared those who did not attended any courses. However, no statistically significant interactions between course attendance and time across all service use variables were observed. Allard et al. (78) also observed a change in employment status after the participation in a RC course, with a number of economically inactive learners reduced from 53 to 19 between pre- and post-course. In larger studies (79, n=463; 80, n=184), statistically significative reductions in emergency room visits, in admissions and in hospitalization days of learners using mental health services were reported. These larger studies also suggested a reduction in healthcare costs. Using a cost-benefit analysis approach, Cronin et al. (80) propose a net cost savings of 269 Australian dollars per student per year.

This fourth qualitative cluster of articles “*Service utilization and cost analysis*”, which will need to be replicated, play an important role in documenting the impact of RCs on reducing the cost and use of health services by learners who use mental health services.

### Qualitative cluster V – status reports (n=8)

Of these eight articles that constitute this qualitative cluster, seven meet the quality criteria of Kmet et al. (27) for qualitative or quantitative research (see **Table 2**). These seven articles present status reports on the state of deployment of the RC, either in a specific country or around the world. Most articles are based on surveys carried out with RC managers or staff. They do not present outcome evaluation data, but rather a portrait of the characteristics of RCs, such as the learners’ characteristics, the fidelity of the original model, the various adaptations, the operational costs and funding (4, 83).

Three articles present a portrait of RCs around the world (3, 4, 83). These articles highlight the rapid growth of RC over the five continents. Overall, two hundred and twenty-two RCs in twenty-eight countries are listed. Despite their wide range of characteristics, most RCs show high levels of fidelity to the original model, particularly for strengths-oriented RCs (4). High fidelity may also be associated with an affiliation of the RC with healthcare agencies or deeply rooted integration in community-based organizations (4, 83). Most RCs scored high overall for their adherence to the following principles: equality, commitment to recovery, being available to all, and being progressive (4). While most RCs are available for anyone, some RCs have been developed to better answer the needs of specific populations, such people with unstable housing, living in forensic settings or coming from LGBTQ+, ethnic and spiritual diverse backgrounds (3, 83). Successful implementation of RC is facilitated by a genuine commitment to transforming health systems and rethinking the prevailing culture on mental health (3).

Two articles examine the characteristics of RC learners in the UK and how they compare with the general and clinical populations (85, 86). Their findings suggest that RC learners are relatively comparable to the general population, supporting the inclusive nature of RCs. However, RCs should offer programming that better engages younger people, those over the age of sixty, men and people who identify as LGBTQ+ community (85, 86). Regarding the elderly population, an article documents how RCs in the UK address dementia in their programming, including contrasting perspectives on how recovery should be framed in the context of dementia (87). Finally, an article explores the evaluation strategy used in RC and recommends a personalized, humanistic and accessible approach (84).

This fifth qualitative cluster of articles “*Status reports*” provides a global perspective on the implementation of RCs throughout the world, as well as a reflection on fidelity to the original model and directions for new developments. These articles pave the way for further international comparative articles on RCs.

## Discussion

The aim of this paper is to provide a chronological and systematic analysis of the state-of-the-art of peer-reviewed studies listed between 2013-2024.

From a chronological perspective, the first RC studies, which began in 2013-2014, focused on implementation lessons as well as perceived benefits and understanding the model’s active ingredients (the first two qualitative clusters), using qualitative designs. The first quantitative outcomes study on the RC was published in 2015 by Sara Meddings and inspired several other researchers (third qualitative cluster) (76). The first cost-benefit analysis studies were published in 2018-2019, in the same years as the international comparison studies (last two qualitative clusters). The first studies and most of the studies reviewed come from the UK (38/64 studies). Other studies come from Canada, Australia, and also from Norway, Denmark, Ireland and Italy. Despite the presence of RC in Asia (n=15; Hong Kong, Japan, Thailand) and Africa (n=2; Uganda) (4), no studies in English or French have been published from Asian or African countries. These data seem consistent with the recovery paradigm emerging in Western countries (15, 89, 90) and the greater presence of RC in these same countries (4).

Over the years, studies have evolved towards higher-quality studies, moving from descriptive survey to qualitative, quantitative or mixed designs meeting Kmet’s criteria. Some studies are distinguished by rigorous analysis (excellent methodological quality according to Kmet criteria) of implementation considerations (29, 30), perceived benefits and model mechanisms of action (41, 42), outcomes (65, 66) and international comparison (83, 84). The outcomes studies have evolved towards studies with greater statistical power, moving from analyses of small groups of research subjects (n<35) to analyses of large groups of research subjects (n>85). Two studies are notable for analyzing over 300 research subjects (67, 79). All outcome studies measure effects on learners of the same RC. Twelve studies are multicenter, including two international ones, and are mainly descriptive or qualitative. To keep pace with this progression, future studies should move to crossover designs and randomized controlled trials (79, 80), and favor multicenter, international studies with high statistical power.

Sixty-four articles divided into five qualitative clusters are presented in the paper, showing the richness and scope of the studies, but also the limitations and challenges to be met in the coming years. The first challenge is probably to continue publishing high-quality studies that further the understanding of the RC practice model. Of the 64 articles published, 46 are of high quality; of these, 11 report outcome evaluation studies with standardized measurement scales. The next steps are to consolidate the measurement of outcomes and their sustainability over time of individual impacts, using models with high statistical power. Many authors recommend common measures for impact evaluation study and the pooling and sharing of data within robust methods designs (4, 29, 33, 67, 79, 84). It will also be necessary to supplement these studies with measures of impact at the organizational and societal levels. Published studies report changes observed in healthcare systems, but without quantitative assessment: partnership and collaboration between organizations, practices based on the recovery paradigm, strengths-based approaches, involvement of service users, etc. Only cost-benefit studies have quantitatively addressed these systemic impacts, and they need to be replicated on a larger scale (80, 81). The effects on communities and societies also need to be measured: attitudes towards mental health, reduction of stigma and discrimination, openness to difference, tolerance of others, inclusive environments, etc. For intervention to be considered evidence-based, the quality of the evidence needs to be increased through additional studies and experimental designs of high methodological quality.

The second challenge is measuring the level of fidelity to the RC practice model. To achieve the desired outcomes, quality implementation must be ensured in a rigorous way, in spite of adaptations to a specific context. Several studies replicate results in specific contexts, underscore the importance of developing a skilled, trained, well-supported and reliable workforce (33, 46, 52). It must be possible to adapt the RC practice model without undermining the mechanisms of action. The fidelity measure currently available only documents the principles and values (7), but without specifying the operations and learning strategies for each of them (91). Future studies must deepen our understanding of the mechanisms of action and identify their concrete operationalization with greater accuracy and precision. Also, future studies must be able to better document the training and continuing professional development processes, as well as the tools offered to trainers and RC staff, to ensure the quality and fidelity of the model. What’s more, the effects on RC trainers and staff involving in working within an RC are not sufficiently assessed, even though they are essential for a better understanding of the impact of change on organizations and societies (34, 44, 53). RC trainers and staff have an important role both in the quality implementation of RC, but also as agents of change in our societies.

The third challenge is concerning the financing of the RC and how to involve a group of partners to engage in its implementation (29). This commitment relies on partners’ understanding of the model and its mechanisms of action, and on discussions to explain potential and actual multi-level impacts (29, 33, 51). The impacts documented in this paper at individual, organizational and societal levels are impressive and demonstrate the need for this type of practical model to guide the transformation of the mental health systems and societies. Health systems must truly move to an approach centered on the empowerment of individuals and communities, where prevention, self-determination, resilience, self-management, increased literacy and adaptive strategies are at the heart of interventions (9, 10, 13–15). Organizations must recreate the conditions needed to thrive and be healthy, create enabling environments where everyone can feel included, foster supportive relationships and openness to others, and reduce exclusion and stigmatizing behaviors (9, 10, 15). The RC practice model, through its multi-level actions and impacts, could act as a pioneering intervention (41). To achieve this, RC must continue to be implemented and studied.

### Limitations

Despite an exhaustive analysis of RC evaluative articles, this article has several limitations. The first limitation of literature reviews is to simply summarize the articles found without critical evaluation or integration. To avoid this situation, qualitative clusters were set up to establish links between articles in the same group. They served to assess the quality of the studies in each group and to highlight certain observations. That said, the analysis could certainly have been more comprehensive and complete, but the intention was to provide a state-of-the-art review of RC studies and to identify their evolution over time and future directions. The second limitation is the possible selective consideration of results for synthesis work. Despite the multi-author analysis, some results may not have been considered and presented. The third limitation is the possible presence of heterogeneous and inconsistent results. In a literature synthesis, the analyst’s bias is to highlight only consistent and homogeneous results, to the detriment of inconsistent ones. Finally, this analysis of the literature after 10 years of implementing of the RC practice model does not allow to conclude on the efficacy of the intervention, but rather to assess the studies published to date and guide future studies.

## Conclusion

This article is the first to provide a comprehensive state-of-the-art literature review of all articles published in peer-reviewed journals and focused on evaluative studies concerning the RC practice model since its conception. The five qualitative clusters proposed show the richness and breadth of the studies. They also point the way to the next stages in the maturity of the field of study. The RC represents a powerful approach to today’s challenges which invite us to live together better and fight stigmatization.

## Data Availability

The original contributions presented in the study are included in the article/**Supplementary Material**. Further inquiries can be directed to the corresponding author.

## Footnotes

1 The descriptive method refers here to papers that present descriptive survey results without any qualitative or statistical analysis.

## Supplementary material

Supplementary Table 1 Qualitative cluster I – Implementation lessons (n=12) (in chronological order).

Supplementary Table 2 Qualitative cluster II – Perceived benefits, experience and active ingredients (n=26) (in chronological order).

Supplementary Table 3 Qualitative cluster III – Evaluated outcomes (n=13) (in chronological order).

Supplementary Table 4 Qualitative cluster IV – Service utilization and cost analysis (n=5) (in chronological order).

Supplementary Table 5 Qualitative cluster V – Status reports (n=8) (in chronological order).
