# Supplementary material for: State-of-the-art literature review of Recovery College evaluative studies between 2013-2024
Source: Front Psychiatry. 2025 Aug 4;16:1584110. doi: 10.3389/fpsyt.2025.1584110 (PMC12358375; doi:10.3389/fpsyt.2025.1584110)
Supplement: Supplementary Table 1 — Qualitative cluster I – Implementation lessons (n=12) (in chronological order). [file Table1.docx]

**Supplementary table 1.**

**Qualitative cluster I – Implementation lessons (n=12) (in chronological order)**

| Authors, date, study data’s country of origin | | Attribute 1.  Types of design  (score using Kmet grids) | Attribute 2.  Number of RCs | | Attribute 3.  Target populations | Attribute 4.  Outcomes |
| --- | --- | --- | --- | --- | --- | --- |
| Less than 75% of Kmet criteria | | | | | | |
| 2013 | Zucchelli and Skinner, 2013  UK | Descriptive method  (8.5/20) | | 1 | All (learners, RC staff, partner organizations) | Implementation lessons |
| 2014 | McGregor et al., 2014  UK | Descriptive method  (12.5/20) | | 1 | All (learners, RC staff, partner organizations) | Implementation lessons |
|  | Meddings, Byrne et al.,  2014  UK | Descriptive method  (14/20) | | 1 | All (learners, RC staff, partner organizations) | Implementation lessons |
| 2016 | Chung et al., 2016  Canada | Descriptive method  (8/20) | | 1 | Specific learners (with mental illness and housing instability) | Implementation lessons in a specific context (housing instability) |
|  | Dunn et al., 2016  UK | Qualitative method (including a survey with descriptive analysis)  (15/20) | | 1 | Diverse learners | Facilitators and barriers to participation |
|  | Frayn et al., 2016  UK | Descriptive method  (12.5/20) | | 1 | All (learners, RC staff, partner organizations) | Implementation lessons in a specific context (secure setting) |
| 2017 | Arbour and Stevens, 2017  Canada | Descriptive method  (7/20) | | 1 | Specific learners (with mental illness) | Implementation lessons |
| More than 76% of Kmet criteria | | | | | | |
| 2018 | Harper and McKeown, 2018  UK | Qualitative method  (16.5/20) | | 1 | Specific learners (with mental illness) | Facilitators and barriers to participation |
|  | Hopkins, Foster et al., 2018  Australia | Qualitative method  (15.5/20) | | 1 | RC staff (RC staff, trainers, managers) | Implementation lessons in a specific context (youth-focused) |
| 2023 | Ali et al.,  2022  UK | Qualitative method  (20/20) | | 1 | All (learners, RC staff, partner organizations) | Implementation lessons |
|  | Andersen et al., 2023  Danemark | Qualitative method  (19.5/20) | | 1 | Diverse learners | Facilitators and barriers to participation |
| 2024 | McPhilbin et al., 2024  UK | Qualitative method  (19/20) | | 31 | RC staff (managers) | Implementation lessons in a specific context (Covid-19 pandemic) |

**Supplementary table 2.**

**Qualitative cluster II – Perceived benefits, experience and active ingredients (n=26) (in chronological order)**

| Authors, date, study data’s country of origin | | Attribute 1.  Types of design  (score using Kmet grids) | Attribute 2.  Number of RCs | Attribute 3.  Target populations | Attribute 4.  Outcomes |
| --- | --- | --- | --- | --- | --- |
| Less than 75% of Kmet criteria | | | | | |
| 2014 | Meddings, Guglietti et al., 2014  UK | Qualitative method (including a survey with descriptive analysis)  (14.5/20) | 1 | Diverse learners | Experience and active ingredients |
| 2015 | Burhouse et al., 2015  UK | Descriptive method  (12/20) | 1 | All (learners, RC staff, partner organizations) | Perceived benefits; Experience and active ingredients |
|  | Skipper et al.,  2015  UK | Descriptive method (narrative account)  (7/20) | 1 | All (learners, RC staff, partner organizations) | Experience and active ingredients |
| 2017 | Windsor et al.,  2017  UK | Qualitative method (with literature review)  (12/20) | 1 | Diverse learners and trainers | Perceived benefits; Experience and active ingredients |
| 2018 | Larsen et al.,  2018  Denmark | Descriptive method (survey)  (8/20) | 1 | Specific learners (mental health professionals) | Perceived benefits |
|  | Lucchi et al.,  2018  Italy | Descriptive method (survey)  (10.5/20) | 1 | All (learners, RC staff, partner organizations) | Experience and active ingredients |
| 2022 | Hopkins et al.,  2022  Australia | Descriptive method (survey)  (14.5/20) | 1 | All (learners, RC staff, partner organizations) | Experience and active ingredients |
| More than 76% of Kmet criteria | | | | | |
| 2016 | Newman-Taylor et al., 2016  UK | Qualitative method  (17/20) | 1 | Diverse learners | Perceived benefits; Experience and active ingredients |
|  | Zabel et al.,  2016  UK | Qualitative method  (19/20) | 1 | Diverse learners | Perceived benefits; Experience and active ingredients |
| 2017 | Perkins et al.,  2017  UK | Qualitative method (including a survey with descriptive analysis)  (17/20) | 1 | Specific learners (corporate, administrative and clinical health system staff) | Perceived benefits; Experience and active ingredients |
| 2018 | Dalgarno et al.,  2018  UK | Qualitative method  (18/20) | 1 | Specific trainers (practitioner trainers) | Experience and active ingredients |
|  | Sommer et al.,  2018  Australia | Qualitative method  (17/20) | 1 | All (learners, RC staff, partner organizations) | Experience and active ingredients |
| 2019 | Crowther et al.,  2019  UK | Qualitative method (with systematic review)  (18/20) | 3 (for the qualitative part) | All (learners, RC staff, partner organizations) | Perceived benefits; Experience and active ingredients |
|  | Dalgarno et al.,  2019  UK | Qualitative method  (19/20) | 1 | Specific trainers (practitioner trainers) | Experience and active ingredients |
|  | Muir-Cochrane et al., 2019  Australia | Qualitative method  (18/20) | 1 | All (learners, RC staff, partner organizations) | Experience and active ingredients |
| 2020 | Khan et al.,  2020  Canada | Qualitative method  (19/20) | 1 | Specific learners (with housing instability) | Experience and active ingredients |
|  | Reid et al.,  2020  Canada | Qualitative method  (17.5/20) | 1 | Specific learners (with housing instability) | Perceived benefits; Experience and active ingredients |
| 2021 | Khan et al.,  2021  Canada | Qualitative method  (19/20) | 1 | Specific learners (with housing instability) and RC staff | Experience and active ingredients |
|  | Oates et al.,  2021  UK | Qualitative method  (18/20) | 1 | Specific trainers (student nurses) | Perceived benefits |
|  | Thompson et al.,  2021  UK | Qualitative method  (18/20) | 1 | Diverse learners | Perceived benefits; Experience and active ingredients |
|  | Briand et al.,  2023  Canada | Qualitative method  (19/20) | 1 | Diverse learners | Perceived benefits |
|  | Doroud et al.,  2023  Australia | Qualitative method (including a survey with descriptive analysis)  (19.5/20) | 1 | All (learners, RC staff, partner organizations) | Experience and active ingredients |
| 2023 | Harris et al., 2023  Canada | Qualitative method  (19/20) | 6 | All (learners, RC staff, partner organizations) | Experience and active ingredients |
|  | Selbekk et al., 2023  Norway | Qualitative method  (20/20) | 2 | Specific learners and trainers (with substance use or mental health issue) | Perceived benefits |
|  | Whitehead et al., 2023  Australia | Qualitative method (including a survey with descriptive analysis) (17.5/20) | 1 | Diverse learners, including youth (12-25 years old) | Experience and active ingredients |
| 2024 | O’Brien et al., 2024  Ireland | Qualitative method (including a survey with descriptive analysis) (16/20) | 4 | All (learners, RC staff, partner organizations) | Perceived benefits; Experience and active ingredients |

**Supplementary table 3.**

**Qualitative cluster III – Evaluated outcomes (n=13) (in chronological order)**

| Authors, date, study data’s country of origin | | Attribute 1.  Types of design  (score using Kmet grids) | Attribute 2.  Number of RCs | Attribute 3.  Target populations | Attribute 4.  Outcomes |
| --- | --- | --- | --- | --- | --- |
| Less than 75% of Kmet criteria | | | | | |
| 2015 | Meddings et al.,  2015  UK | Mixed method  (17/28) | 1 | Specific learners (with mental illness) | Evaluated outcomes |
| 2017 | Nurser et al.,  2017  UK | Mixed method  (15.5/28) | 1 | Diverse learners | Evaluated outcomes |
| More than 76% of Kmet criteria | | | | | |
| 2018 | Ebrahim et al.,  2018  UK | Mixed method  (22/28) | 1 | Diverse learners | Evaluated outcomes |
|  | Hopkins, Pedwell et al.,  2018  Australia | Mixed method (home survey)  (26/28) | 1 | Diverse learners | Evaluated outcomes |
|  | Stevens et al.,  2018  UK | Mixed method  (22/28) | 1 | Specific learners (with mental illness) | Evaluated outcomes |
| 2019 | Sommer et al.,  2019  Australia | Quantitative method  (23/28) | 1 | Diverse learners | Evaluated outcomes |
|  | Sutton & French,  2019  UK | Quantitative method  (24/28) | 1 | Specific learners (health professionals) | Considerations for evaluated outcomes |
|  | Wilson et al.,  2019  UK | Mixed method  (24/28) | 1 | Specific learners (with mental illness) | Evaluated outcomes |
| 2021 | Durbin et al.,  2021  Canada | Quantitative method  (28/28) | 1 | Specific learners (with mental illness and housing instability) | Evaluated outcomes |
| 2022 | Rapisarda et al.,  2022  Canada | Quantitative method  (27.5/28) | 1 | Diverse learners | Evaluated outcomes |
|  | Yoeli et al.,  2022  UK | Mixed method  (26.5/28) | 1 | Diverse learners | Evaluated outcomes |
| 2024 | Briand et al.,  2024  Canada | Quantitative method  (26/28) | 1 | Diverse learners | Evaluated outcomes |
|  | Paul et al.,  2024  Canada | Mixed method  (25/28) | 1 | Specific learners (postsecondary students) | Evaluated outcomes |

**Supplementary table 4.**

**Qualitative cluster IV – Service utilization and cost analysis (n=5) (in chronological order)**

| Authors, date, study data’s country of origin | | Attribute 1.  Types of design  (score using Kmet grids) | Attribute 2.  Number of RCs | Attribute 3.  Target populations | Attribute 4.  Outcomes |
| --- | --- | --- | --- | --- | --- |
| Less than 75% of Kmet criteria | | | | | |
| 2019 | Kay et al.,  2019  UK | Descriptive method  (7/28) | 1 | Diverse learners | Services use and potential cost savings |
| More than 76% of Kmet criteria | | | | | |
| 2018 | Bourne et al.,  2018  UK | Quantitative method  (26/28) | 1 | Specific learners (with mental illness)(n=463) | Services use and potential cost savings |
| 2019 | Sutton, Lawrence et al., 2019  UK | Quantitative method  (23/28) | 1 | Specific learners (with mental illness) | Services use |
| 2021 | Cronin et al.,  2021  Australia | Quantitative method  (26/28) | 1 | Specific learners (with mental illness)(n=184) | Services use and cost-benefit |
| 2024 | Allard et al., 2024  UK | Quantitative method  (26.5/28) | 1 | Diverse learners (n=84) | Service use and evaluated outcomes |

**Supplementary Table 5.**

**Qualitative cluster V – Status reports (n=8) (in chronological order)**

| Authors, date, study data’s country of origin | | Attribute 1.  Types of design  (score using Kmet grids) | Attribute 2.  Number of RCs | Attribute 3.  Key informants | Attribute 4.  Outcomes |
| --- | --- | --- | --- | --- | --- |
| Less than 75% of Kmet criteria | | | | | |
| 2019 | Lowen et al.,  2019  UK | Descriptive method (survey)  (14.5/20) | 11 | RC managers | Status report |
| More than 76% of Kmet criteria | | | | | |
| 2019 | King et al.,  2019  International | Descriptive method (survey)  (15.5/20) | 77 | RC respondents (likely managers) | Status report |
|  | Meddings et al., 2019  UK | Quantitative method  (22/28) | 1 | Diverse learners | Status report |
| 2023 | Bowness et al.,  2023  UK | Quantitative method  (27/28) | 3 | Specific learners (mental health service users) | Status report |
|  | Hayes, Camacho et al.,  2023  UK | Quantitative method (survey)  (28/28) | 63 | RC managers | Status report costs |
|  | Hayes, Hunter-Brown et al.,  2023  International | Quantitative method (survey)  (27/28) | 221 | RC managers | Status report costs |
|  | Soklaridis et al.,  2023  Canada | Qualitative method  (19.5/20) | Unknown (but many) | RC staff and partners organization | Status report |
|  | Wolverson et al.,  2023  UK | Qualitative method (including a survey with descriptive analysis)  (16/20) | 51 | RC staff and partners organization | Status report |
